# Crystal structures of three lead(II) acetate-bridged di­amino­benzene coordination polymers

**DOI:** 10.1107/S1600536814025380

**Published:** 2014-11-21

**Authors:** David K. Geiger, Dylan E. Parsons, Patricia L. Zick

**Affiliations:** a Department of Chemistry, SUNY-College at Geneseo, Geneseo, NY 14454, USA

**Keywords:** crystal structure, coordination polymer, lead(II), hydrogen bonds, benzene-1,2-di­amine, hemidirected coordination

## Abstract

Poly[tris­(acetato-κ^2^ *O*,*O*′)(μ_2_-acetato-κ^3^ *O*,*O*′:*O*)tetra­kis­(μ_3_-acetato-κ^4^ *O*,*O*′:*O*:*O*′)bis­(benzene-1,2-di­amine-κ*N*)tetra­lead(II)], [Pb_4_(CH_3_COO)_8_(C_6_H_8_N_2_)_2_]_*n*_, (I), poly[(acetato-κ^2^ *O*,*O*′)(μ_3_-acetato-κ^4^ *O*,*O*′:*O*:*O*′)(4-chloro­benzene-1,2-diamine-κ*N*)lead(II)], [Pb(CH_3_COO)_2_(C_6_H_7_ClN_2_)]_*n*_, (II), and poly[(κ^2^ *O*,*O*′)(μ_3_-acetato-κ^4^ *O*,*O*′:*O*:*O*′)(3,4-di­amino­benzo­nitrile-κ*N*)lead(II)], [Pb(CH_3_COO)_2_(C_7_H_7_N_3_)]_*n*_, (III), have polymeric structures in which monomeric units are joined by bridging acetate ligands. All of the Pb^II^ ions exhibit hemidirected coordination. The repeating unit in (I) is composed of four Pb^II^ ions having O_6_, O_6_N, O_7_ and O_6_N coordination spheres, respectively, where N represents a monodentate benzene-1,2-di­amine ligand and O acetate O atoms. Chains along [010] are joined by bridging acetate ligands to form planes parallel to (10-1). (II) and (III) are isotypic and have one Pb^II^ ion in the asymmetric unit that has an O_6_N coordination sphere. Pb_2_O_2_ units result from a symmetry-imposed inversion center. Polymeric chains parallel to [100] exhibit hydrogen bonding between the amine and acetate ligands. In (III), additional hydrogen bonds between cyano groups and non-coordinating amines join the chains by forming *R*_2_^2^(14) rings.

## Abstract 2

The structures of three lead(II) coordination polymers are reported. One exhibits a two-dimensional structure, whereas the other two are one-dimensional. All three exhibit bidentate bridging acetate and monodentate benzene-1,2-di­amine ligands. The extended structures reveal extensive hydrogen-bonding networks involving the di­amine and acetate ligands.

## Chemical context   

Metal–organic frameworks (MOFs) are of inherent inter­est in areas such as gas storage, catalysis, chemical sensors and mol­ecular separation (Dey *et al.*, 2014 ▶; Kreno *et al.*, 2012 ▶; Farha & Hupp, 2010 ▶). Recently, we reported the synthesis and structural characterization of two zinc MOFs possessing bridging acetate ligands and monodentate chloro- or cyano-substituted *o*-phenyl­enedi­amine ligands (Geiger & Parsons, 2014 ▶). These complexes possess a ladder–chain structure with an ethanol mol­ecule that occupies a void with a volume of approximately 224 Å^3^. The results presented here expand the structural study to Pb^II^ analogues.

Pb^II^ compounds often exhibit a distorted coordination sphere or open coordination site that has been attributed to stereoactive ‘lone-pair’ electrons (Morsali, 2004 ▶; Wang & Liebau, 2007 ▶; Park & Barbier, 2001 ▶). Indeed, hemidirected geometry is favored over halodirected geometry for Pb^II^ when hard ligands are present, which corresponds to a greater ionic character in the metal–ligand bonding (Shimoni-Livny *et al.*, 1998 ▶), or when one or more of the ligands is anionic (Esteban-Gómez *et al.*, 2006 ▶). However, hemidirected lead(II) complexes in a soft sulfur-rich environment are also known (Imran *et al.*, 2014 ▶). The results of a reduced variational space (RVS) analysis suggest that more sterically crowded, hemidirected structures are stabilized by polarization of the lead(II) ion induced by the ligand arrangement (Devereux *et al.*, 2011 ▶). The possibility of a distorted coordination sphere enhancing the volume of void space between chains found in coordination polymers provided the impetus for the synthesis and structural characterization of the compounds reported herein.
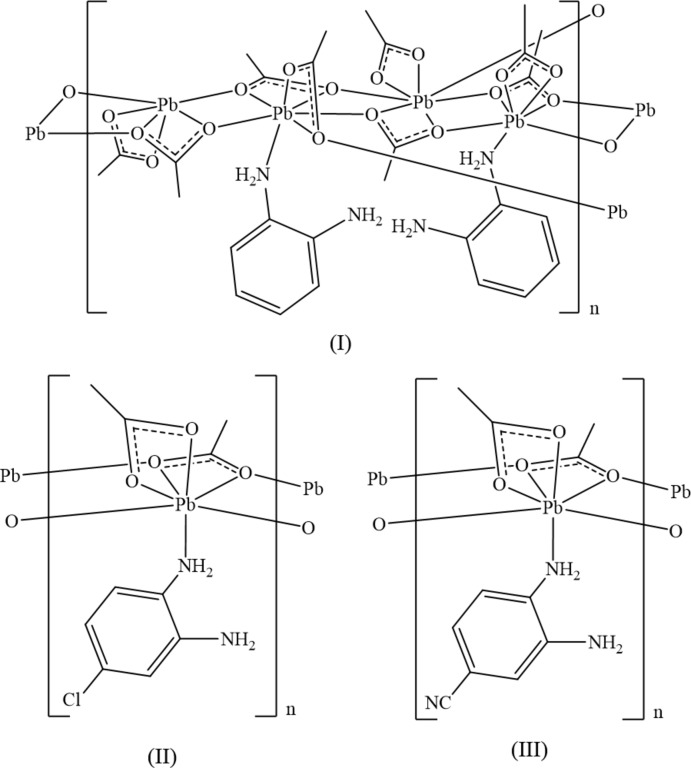


## Structural commentary   

**Figure 1:**
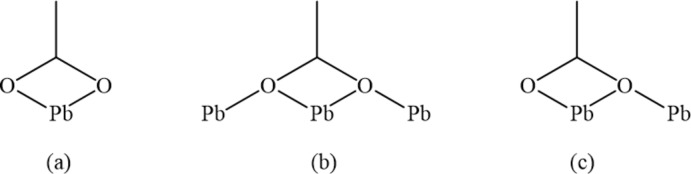
The three acetate coordination modes observed in (I), (II), and (III), showing (*a*) acetato-κ^2^ *O*,*O*′, (*b*) μ_3_-acetato-κ^4^ *O*,*O*′:*O*:*O*′, and (*c*) μ_2_-acetato-κ^3^ *O*,*O*′:*O*.

**Figure 2:**
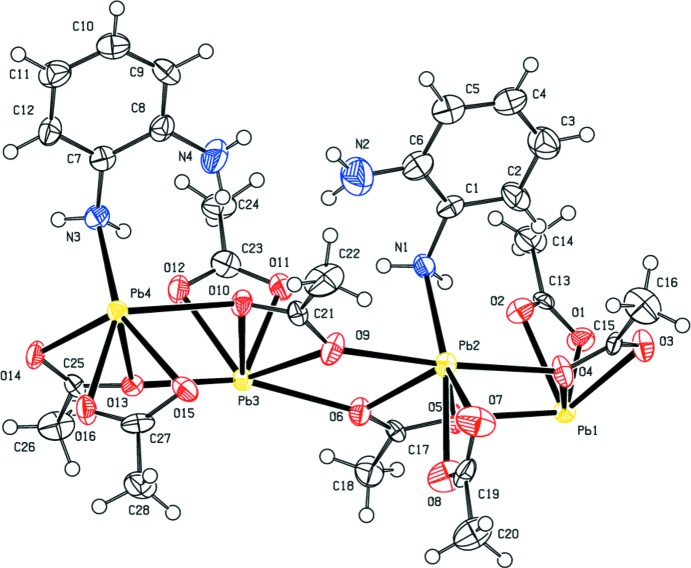
The atom-labeling scheme for (I). Anisotropic displacement parameters are drawn at the 50% probability level.

Fig. 1 ▶ shows the three acetate coordination modes displayed by (I), (II), and (III). The three modes will be referred to hereafter as types (*a*), (*b*) and (*c*). As seen in Fig. 2 ▶, the asymmetric unit of (I) has four symmetry-independent Pb atoms. The Pb atoms are linked by bridging acetate ligands of type (*b*) to form a ladder-chain parallel to [010]. Each is also coordinated to a bidentate acetate ligand of type (*a*) and Pb2 and Pb4 have an amine nitro­gen in their coordination spheres. Finally, atoms Pb3 and Pb4 are linked by an acetato ligand of type (*c*). The two benzene-1,2-di­amine ligands are approximately coplanar. The angle formed by the benzene mean planes is 6.1 (4)°, with N1, N2, N3 and N4 being 0.051 (16), 0.013 (19), 0.074 (16), and 0.034 (16) Å from their respective planes.

**Figure 3:**
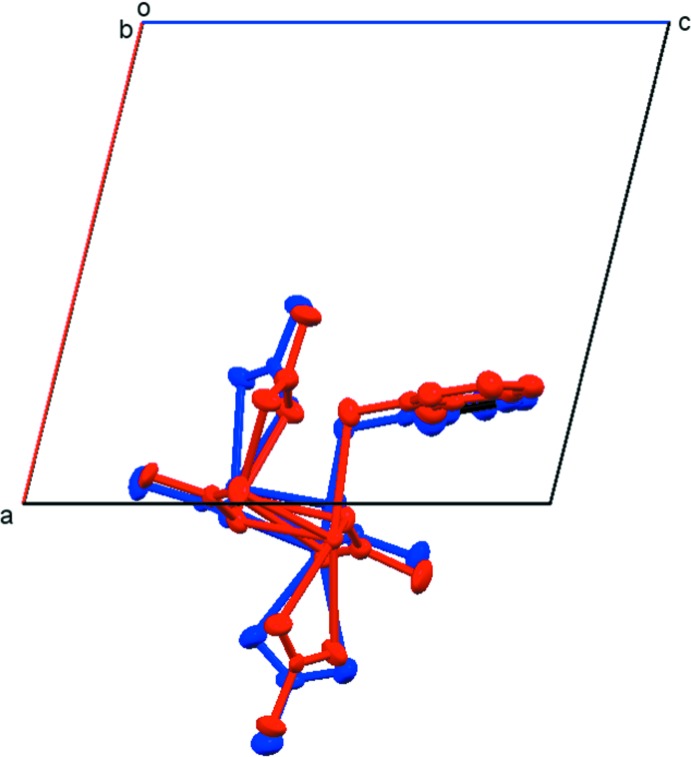
A view of (I) in which the two halves of the asymmetric unit related by the pseudo-translation are color coded. H atoms have been omitted for clarity.

**Figure 4:**
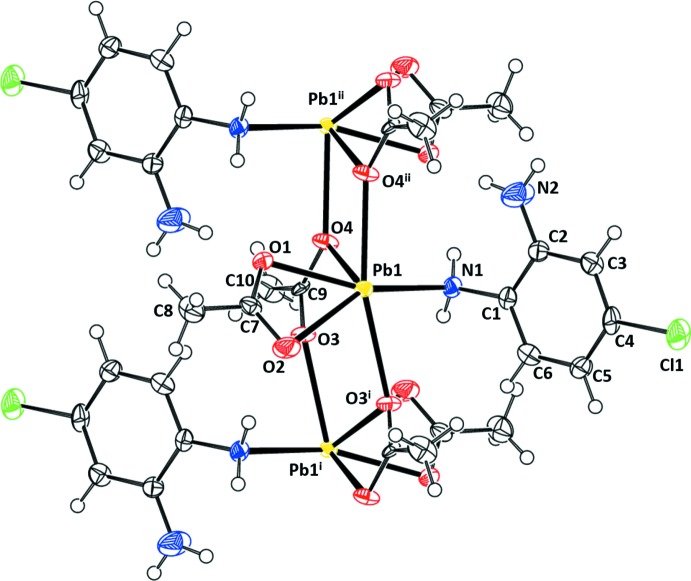
The atom-labeling scheme for (II). Anisotropic displacement parameters are drawn at the 50% probability level. [Symmetry identifiers: (i) −*x* + 1, −*y*, −*z* + 1; (ii) −*x* + 2, −*y*, −*z* + 1.]

**Figure 5:**
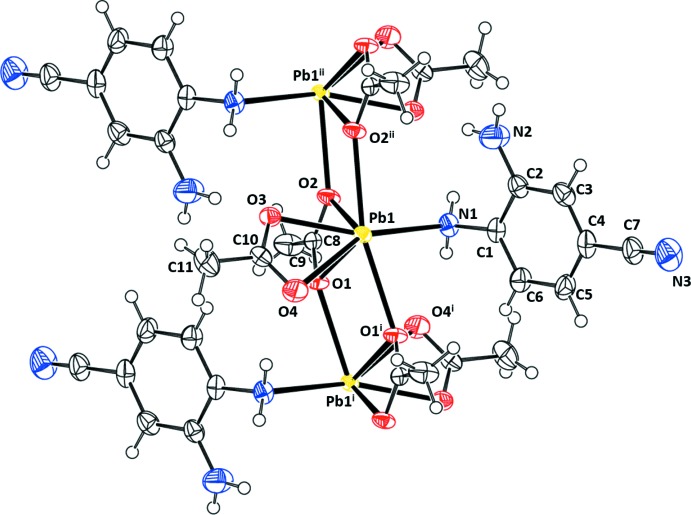
The atom-labeling scheme for (III). Anisotropic displacement parameters are drawn at the 50% probability level. [Symmetry identifiers: (i) −*x* + 1, −*y*, −*z* + 1; (ii) −*x* + 2, −*y*, −*z* + 1.]

The asymmetric unit of (I) possesses pseudo-translational symmetry as a result of the similarity in the coordination geometries exhibited by Pb1 and Pb3 and by Pb2 and Pb4. Pb1⋯Pb3 = 7.4548 (10) Å and Pb2⋯Pb4 = 7.5372 (10) Å, approximately half of the Pb1⋯Pb4^i^ = 14.989 (2) Å distance (see Table 1 ▶ for symmetry codes). Fig. 3 ▶ shows a representation of (I) in which the two pseudo-translationally related halves of the asymmetric unit are color coded. Primary differences in the two halves involve the orientation of the two non-coordinating amine groups, one less acetate type (*c*) on Pb1 than on Pb3, and a type (*c*) acetate ligand on Pb2 replaced by a type (*a*) acetate ligand on Pb4.

(II) and (III) are isotypic if the nitrile function in (III) is considered as a large one-atomic group and replaces the Cl atom in (II). Fig. 4 ▶ shows the atom-labeling scheme for (II) and Fig. 5 ▶ shows the atom-labeling scheme for (III). Each Pb atom has two bidentate acetate ligands, one of type (*a*) and one of type (*b*). The type (*b*) ligands result in chains parallel to [100], with Pb_2_O_2_ cores related by inversion centers. The substituted benzene-1,2-di­amine ligands are essentially planar. For (II), N1 and N2 are below the plane by 0.056 (14) and 0.066 (18) Å, respectively, and Cl1 is 0.020 (14) Å above the plane. In (III), N1 and N2 are 0.073 (17) and 0.05 (2) Å out of the plane. The C7—N3—C4 angle is 177.7 (16)° and N3 is 0.12 (2) Å out of the plane.

**Figure 6:**
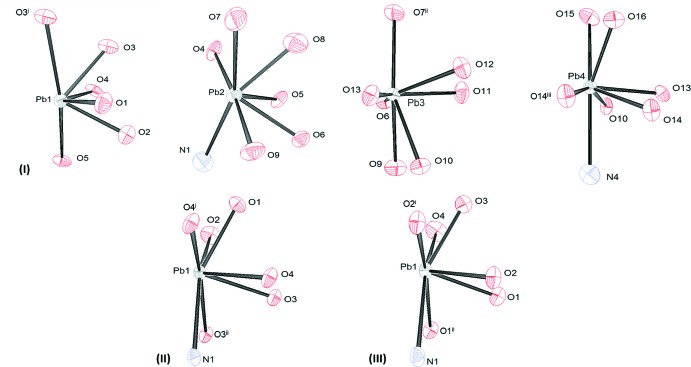
Representation of the Pb^II^ coordination environments observed in (I), (II), and (III). Symmetry identifiers are those used in Tables 1 ▶, 2 ▶ and 3 ▶.

The coordination spheres are O_6_, O_6_N, O_7_, and O_6_N for Pb1, Pb2, Pb3, and Pb4, respectively, for (I), and O_6_N for (II) and (III). Representations of the coordination spheres are shown in Fig. 6 ▶ and pertinent bond distances are found in Tables 1 ▶, 2 ▶ and 3 ▶. The coordination is clearly hemidirected for each Pb and the Pb—O bond lengths are asymmetrical, as is often found for hemidirected compounds (Shimoni-Livny *et al.*, 1998 ▶). The average Pb—O bond lengths are 2.60 (13), 2.59 (11), and 2.58 (12) Å for (I), (II) and (III), respectively, or 2.59 (12) Å overall, and range from 2.380 (6) to 2.901 (6) Å. The average Pb—N bond length for the three compounds is 2.84 (5) Å. In all cases, the Pb—O(N) bond lengths are longer for those ligand atoms adjacent to the open coordination site. This is consistent with structural results for other hemidirected coordination modes involving O- and N-donor atoms (*cf.* Shimoni-Livny *et al.*, 1998 ▶; Morsali *et al.*, 2005 ▶; Esteban-Gómez *et al.*, 2006 ▶; Morsali, 2004 ▶).

## Supra­molecular features   

**Figure 7:**
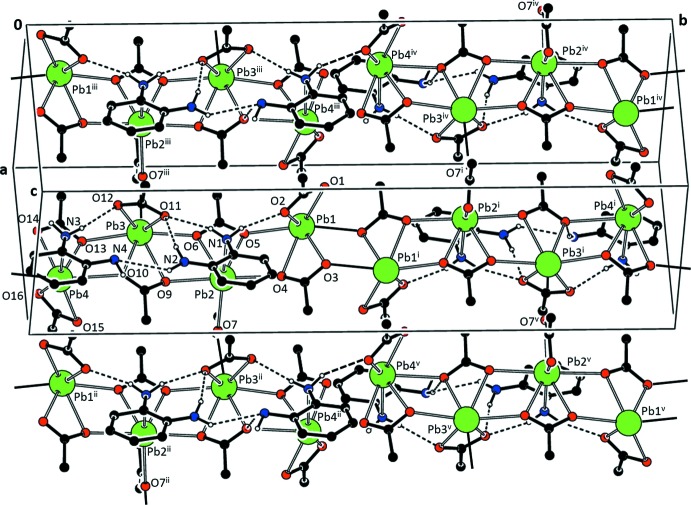
Packing diagram for (I), showing the linked chains. Hydrogen bonds are represented by dashed lines. H atoms not involved in the hydrogen-bonding network are not shown. [Symmetry identifiers: (i) −*x* + 2, −*y* + 1, −*z* + 1; (ii) *x* + 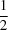, *y* + 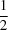, *z* + 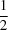; (iii) *x* − 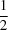, −*y* + 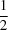, *z* − 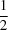; (iv) −*x* + 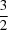, *y* + 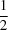, −*z* + 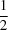; (v) −*x* + 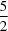, *y* + 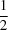, −*z* + 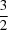.]

The one-dimensional asymmetric unit chain of (I) propagates *via* inversion centers and is extended into two dimensions *via* an acetate ligand of type (*c*) that bridges Pb2 and Pb3^iii^, as shown in Fig. 7 ▶, where the symmetry designators are defined. The result is an extended structure composed of planes parallel to (10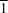). N—H⋯O and N—H⋯N hydrogen bonding is observed along the chains parallel to [010] (see Table 4 ▶).

**Figure 8:**
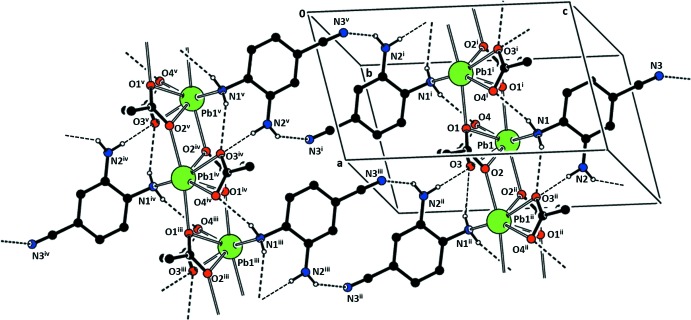
Packing diagram for (III), showing the chains joined by N—H⋯N C hydrogen bonds. Hydrogen bonds are represented by by dashed lines. H atoms not involved in the hydrogen-bonding network are not shown. [Symmetry identifiers: (i) −*x* + 1, −*y* + 1, −*z* + 1; (ii) −*x* + 2, −*y* + 1, −*z* + 1; (iii) *x* + 1, *y* − 1, *z* − 1; (iv) −*x* + 2, −*y*, −*z*; (v) *x*, *y* − 1, *z* − 1.]

In compounds (II) and (III), chains parallel to [100] are observed. An extensive N—H⋯O hydrogen-bonding network is found along the chains (see Tables 5 ▶ and 6 ▶). For (III), the nitrile group affords the opportunity for additional hydrogen bonding. As seen in Fig. 8 ▶, this results in 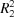
(14) rings involving N—H⋯N C hydrogen bonds between adjacent chains.

Based on calculations performed with *PLATON* (Spek, 2009 ▶), no solvent-accessible voids are found in (I), (II), or (III).

## Database survey   

Numerous examples of polymeric lead(II) compounds with bridging carboxyl­ate ligands possessing a range of coordination modes have been reported (for examples, see Lyczko & Bak, 2008 ▶; Dai *et al.*, 2009 ▶; Mohammadnezhad *et al.*, 2010 ▶; Yilmaz *et al.*, 2003 ▶; Foreman *et al.*, 2001 ▶). A zinc metal organic framework with bridging acetate ligands and a monodentate 4-chloro­benzene-1,2-di­amine ligand has been reported (Geiger & Parsons, 2014 ▶).

## Synthesis and crystallization   

### Preparation of (I)   

Benzene-1,2-di­amine (0.109 g, 0.93 mmol) was stirred into a solution of lead(II) acetate trihydrate (0.175 g, 0.46 mmol) in ethanol (10 ml). The solution was heated to a gentle reflux for 2 h and then cooled to room temperature. The solvent was reduced in volume by slow evaporation. After 5 d, crystals suitable for X-ray analysis had formed. Further solvent reduction resulted in precipitation of excess di­amine, so the overall yield was not determined. Selected IR bands (diamond anvil, cm^−1^): 3353 (*br*), 1505 (*s*), 1932 (*s*), 1284 (*s*) 1045 (*w*), 1018 (*w*), 939 (*w*).

### Preparation of (II)   

4-Chloro­benzene-1,2-di­amine (0.106 g, 0.75 mmol) was dissolved in boiling ethanol (10 ml) and lead(II) acetate trihydrate (0.134 g, 0.35 mmol) was added with stirring. The resulting solution was refluxed for 4 h, removed from the heat and the solvent was allowed to slowly evaporate. The residue obtained was dissolved in hot methanol and passed through a 45 µm pore filter. Crystals suitable for X-ray analysis were obtained after slow evaporation of the solvent. Further solvent reduction resulted in precipitation of excess di­amine and so the overall yield was not determined. Selected IR bands (diamond anvil, cm^−1^): 3334 (*br*), 1537 (*s*), 1393 (*s*), 1337 (*s*), 1018 (*s*).

### Preparation of (III)   

To a solution of lead(II) acetate trihydrate (0.149 g, 0.39 mmol) in ethanol (10 ml) was added 3,4-di­amino­benzo­nitrile (0.104 g, 0.75 mmol). The resulting solution was stirred at a gentle reflux for 1 h. The solvent was allowed to slowly evaporate over a period of 3 d, resulting in crystals suitable for X-ray analysis. Further solvent reduction resulted in precipitation of excess di­amine and so the overall yield was not determined. Selected IR bands (diamond anvil, cm^−1^): 3432 (*w*), 3316 (*w*), 2213 (*s*), 1581 (*s*), 1557 (*s*), 1394 (*s*), 1301 (*s*), 1020 (*s*).

## Refinement   

Crystal data, data collection and structure refinement details are summarized in Table 7 ▶. All H atoms were observed in difference Fourier maps. C-bonded H atoms were refined using a riding model, with C—H = 0.98 Å for the methyl groups and 0.95 Å for the aromatic ring. The C—H hydrogen isotropic displacement parameters were fixed using the approximation *U*_iso_(H) = 1.5*U*_eq_(C) for the methyl H atoms and 1.2*U*_eq_(C) for the aromatic H atoms. The atomic coordinates for the amine H atoms were refined using an N—H bond-distance restraint of 0.88 (2) Å and the H-atom isotropic displacement parameters were set using the approximation *U*_iso_(H) = 1.5*U*_eq_(N). Late in the refinement, a correction for extinction was applied for each of the structures. For (I), the highest residual electron-density peak is 0.94 Å from Pb2 and the deepest hole is 1.20 Å from Pb3. The highest residual electron-density peak is 0.89 Å and the deepest hole is 0.91 Å from Pb1 in (II). For (III), the highest residual electron-density peak and the deepest hole are 0.92 Å and 0.82 Å, respectively, from Pb1.

## Supplementary Material

Crystal structure: contains datablock(s) global, I, II, III. DOI: 10.1107/S1600536814025380/zl2608sup1.cif

Structure factors: contains datablock(s) I. DOI: 10.1107/S1600536814025380/zl2608Isup2.hkl

Supporting information file. DOI: 10.1107/S1600536814025380/zl2608Isup5.mol

Structure factors: contains datablock(s) II. DOI: 10.1107/S1600536814025380/zl2608IIsup3.hkl

Supporting information file. DOI: 10.1107/S1600536814025380/zl2608IIsup6.mol

Structure factors: contains datablock(s) III. DOI: 10.1107/S1600536814025380/zl2608IIIsup4.hkl

Supporting information file. DOI: 10.1107/S1600536814025380/zl2608IIIsup7.mol

CCDC references: 1035089, 1035088, 1035087

Additional supporting information:  crystallographic information; 3D view; checkCIF report

## Figures and Tables

**Table 1:** Selected bond lengths () for (I)

Pb1O2	2.380(6)	Pb3O12	2.509(7)
Pb1O3	2.474(7)	Pb3O10	2.590(6)
Pb1O4	2.576(7)	Pb3O9	2.604(7)
Pb1O1	2.636(7)	Pb3O7^ii^	2.675(7)
Pb1O5	2.667(6)	Pb3O13	2.688(6)
Pb1O3^i^	2.792(7)	Pb3O6	2.696(7)
Pb2O8	2.448(8)	Pb4O16	2.427(7)
Pb2O6	2.470(7)	Pb4O13	2.482(7)
Pb2O5	2.485(6)	Pb4O14	2.563(7)
Pb2O9	2.654(7)	Pb4O14^iii^	2.609(7)
Pb2O4	2.696(6)	Pb4O15	2.713(7)
Pb2O7	2.747(8)	Pb4O10	2.901(6)
Pb2N1	2.797(9)	Pb4N3	2.862(10)
Pb3O11	2.443(7)		

Symmetry codes: (i) 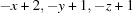; (ii) 
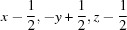
; (iii) 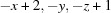.

**Table 2:** Selected bond lengths () for (II)

Pb1O1	2.467(6)	Pb1O2	2.678(7)
Pb1O3	2.504(6)	Pb1O3^ii^	2.734(6)
Pb1O4	2.512(6)	Pb1N1	2.800(8)
Pb1O4^i^	2.632(6)		

Symmetry codes: (i) 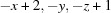; (ii) 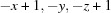.

**Table 3:** Selected bond lengths () for (III)

Pb1O3	2.431(7)	Pb1O4	2.667(8)
Pb1O2	2.485(8)	Pb1O1^ii^	2.727(7)
Pb1O1	2.505(7)	Pb1N1	2.906(10)
Pb1O2^i^	2.635(7)		

Symmetry codes: (i) 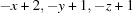; (ii) 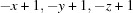.

**Table 4:** Hydrogen-bond geometry (, ) for (I)

*D*H*A*	*D*H	H*A*	*D* *A*	*D*H*A*
N1H1*A*O2	0.89(2)	2.20(3)	3.061(11)	163(7)
N1H1*B*O11	0.88(2)	2.25(4)	3.079(11)	156(8)
N2H2*A*O11	0.90(2)	2.38(5)	3.238(13)	159(11)
N2H2*B*N4	0.90(2)	2.57(5)	3.229(14)	131(5)
N3H3*A*O12	0.87(2)	2.32(4)	3.151(11)	160(8)
N3H3*B*O16^iii^	0.88(2)	2.33(4)	3.145(11)	154(7)
N4H4*B*O10	0.88(2)	2.38(4)	3.236(12)	163(10)

Symmetry code: (iii) 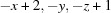.

**Table 5:** Hydrogen-bond geometry (, ) for (II)

*D*H*A*	*D*H	H*A*	*D* *A*	*D*H*A*
N1H1*A*O2^ii^	0.88(2)	2.38(3)	3.261(11)	172(9)
N1H1*B*O1^i^	0.88(2)	2.39(5)	3.201(10)	153(8)
N2H2*A*O1^i^	0.87(2)	2.19(6)	2.998(11)	155(13)

Symmetry codes: (i) 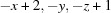; (ii) 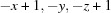.

**Table 6:** Hydrogen-bond geometry (, ) for (III)

*D*H*A*	*D*H	H*A*	*D* *A*	*D*H*A*
N1H1*A*O4^ii^	0.88(2)	2.46(4)	3.310(14)	164(12)
N1H1*B*O3^i^	0.87(2)	2.40(8)	3.139(12)	143(11)
N2H2*A*O3^i^	0.88(2)	2.25(9)	3.044(14)	150(16)
N2H2*B*N3^iii^	0.88(2)	2.62(11)	3.355(18)	142(14)

Symmetry codes: (i) 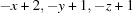; (ii) 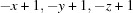; (iii) 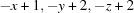.

**Table 7:** Experimental details

	(I)	(II)	(III)
Crystal data			
Chemical formula	[Pb_4_(C_2_H_3_O_2_)_8_(C_6_H_8_N_2_)_2_]	[Pb(C_2_H_3_O_2_)_2_(C_6_H_7_ClN_2_)]	[Pb(C_2_H_3_O_2_)_2_(C_7_H_7_N_3_)]
*M*_r_	1517.40	467.86	458.43
Crystal system, space group	Monoclinic, *P*2_1_/*n*	Triclinic, *P* 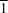	Triclinic, *P* 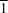
Temperature (K)	200	200	200
*a*, *b*, *c* ()	11.1447(14), 29.694(4), 11.8597(14)	7.3623(10), 7.6177(10), 13.1413(17)	7.3724(8), 7.6349(8), 13.4069(15)
, , ()	90, 103.941(4), 90	89.762(4), 76.405(4), 66.691(4)	88.839(3), 78.330(3), 66.035(3)
*V* (^3^)	3809.1(8)	654.63(15)	673.71(13)
*Z*	4	2	2
Radiation type	Mo *K*	Mo *K*	Mo *K*
(mm^1^)	17.70	13.10	12.54
Crystal size (mm)	0.50 0.30 0.10	0.30 0.10 0.10	0.30 0.20 0.05

Data collection			
Diffractometer	Bruker SMART X2S benchtop	Bruker SMART X2S benchtop	Bruker SMART X2S benchtop
Absorption correction	Multi-scan (*SADABS*; Bruker, 2013 ▶)	Multi-scan (*SADABS*; Bruker, 2013 ▶)	Multi-scan (*SADABS*; Bruker, 2013 ▶)
*T*_min_, *T*_max_	0.12, 0.27	0.11, 0.35	0.12, 0.57
No. of measured, independent and observed [*I* > 2(*I*)] reflections	26670, 7708, 5239	6572, 2572, 2262	8223, 2819, 2498
*R*_int_	0.080	0.057	0.053
(sin /)_max_ (^1^)	0.624	0.625	0.641

Refinement			
*R*[*F* ^2^ > 2(*F* ^2^)], *wR*(*F* ^2^), *S*	0.042, 0.093, 0.96	0.038, 0.100, 1.04	0.041, 0.145, 1.14
No. of reflections	7708	2572	2819
No. of parameters	502	178	187
No. of restraints	122	6	96
H-atom treatment	H atoms treated by a mixture of independent and constrained refinement	H atoms treated by a mixture of independent and constrained refinement	H atoms treated by a mixture of independent and constrained refinement
_max_, _min_ (e ^3^)	2.12, 1.97	3.38, 3.08	3.41, 2.68

Computer programs: *APEX2* and *SAINT* (Bruker, 2013 ▶), *SHELXS97* and *SHELXL2014* (Sheldrick, 2008 ▶), *PLATON* (Spek, 2009 ▶), *Mercury* (Macrae *et al.*, 2006 ▶), *ORTEP-3 for Windows* (Farrugia, 2012 ▶) and *publCIF* (Westrip, 2010 ▶).

## supplementary crystallographic information

### Crystal data

**Table:**

[Pb(C_2_H_3_O_2_)_2_(C_7_H_7_N_3_)]	*Z* = 2
*M**_r_* = 458.43	*F*(000) = 428
Triclinic, *P*1	*D*_x_ = 2.260 Mg m^−^^3^
*a* = 7.3724 (8) Å	Mo *K*α radiation, λ = 0.71073 Å
*b* = 7.6349 (8) Å	Cell parameters from 117 reflections
*c* = 13.4069 (15) Å	θ = 3.4–27.1°
α = 88.839 (3)°	µ = 12.54 mm^−^^1^
β = 78.330 (3)°	*T* = 200 K
γ = 66.035 (3)°	Plate, clear colourless
*V* = 673.71 (13) Å^3^	0.30 × 0.20 × 0.05 mm

### Data collection

**Table:**

Bruker SMART X2S benchtop diffractometer	2819 independent reflections
Radiation source: sealed microfocus tube	2498 reflections with *I* > 2σ(*I*)
Doubly curved silicon crystal monochromator	*R*_int_ = 0.053
ω scans	θ_max_ = 27.1°, θ_min_ = 2.9°
Absorption correction: multi-scan (*SADABS*; Bruker, 2013)	*h* = −9→9
*T*_min_ = 0.12, *T*_max_ = 0.57	*k* = −9→9
8223 measured reflections	*l* = −17→17

### Refinement

**Table:**

Refinement on *F*^2^	Secondary atom site location: difference Fourier map
Least-squares matrix: full	Hydrogen site location: mixed
*R*[*F*^2^ > 2σ(*F*^2^)] = 0.041	H atoms treated by a mixture of independent and constrained refinement
*wR*(*F*^2^) = 0.145	*w* = 1/[σ^2^(*F*_o_^2^) + (0.0965*P*)^2^] where *P* = (*F*_o_^2^ + 2*F*_c_^2^)/3
*S* = 1.14	(Δ/σ)_max_ < 0.001
2819 reflections	Δρ_max_ = 3.41 e Å^−^^3^
187 parameters	Δρ_min_ = −2.68 e Å^−^^3^
96 restraints	Extinction correction: *SHELXL2014* (Sheldrick, 2008)
Primary atom site location: structure-invariant direct methods	Extinction coefficient: 0.0029 (14)

### Special details

**Table:**

Geometry. All e.s.d.'s (except the e.s.d. in the dihedral angle between two l.s. planes) are estimated using the full covariance matrix. The cell e.s.d.'s are taken into account individually in the estimation of e.s.d.'s in distances, angles and torsion angles; correlations between e.s.d.'s in cell parameters are only used when they are defined by crystal symmetry. An approximate (isotropic) treatment of cell e.s.d.'s is used for estimating e.s.d.'s involving l.s. planes.

### Fractional atomic coordinates and isotropic or equivalent isotropic displacement parameters (Å^2^)

**Table:**

	*x*	*y*	*z*	*U*_iso_*/*U*_eq_	
Pb1	0.67380 (5)	0.66477 (5)	0.53022 (2)	0.0203 (2)	
N1	0.7227 (14)	0.4007 (13)	0.6920 (7)	0.0275 (19)	
H1A	0.670 (16)	0.320 (12)	0.682 (11)	0.041*	
H1B	0.845 (8)	0.333 (14)	0.702 (10)	0.041*	
N2	0.8921 (17)	0.592 (2)	0.8005 (10)	0.059 (4)	
H2A	0.97 (2)	0.54 (2)	0.742 (7)	0.088*	
H2B	0.94 (2)	0.67 (2)	0.823 (12)	0.088*	
C1	0.6114 (17)	0.5081 (16)	0.7846 (8)	0.027 (2)	
C2	0.6936 (17)	0.6094 (15)	0.8374 (8)	0.028 (2)	
C3	0.5729 (17)	0.7188 (17)	0.9252 (9)	0.036 (3)	
H3	0.6284	0.7802	0.9635	0.043*	
C4	0.3739 (18)	0.7430 (18)	0.9601 (9)	0.035 (2)	
C5	0.2911 (18)	0.6475 (18)	0.9083 (9)	0.038 (3)	
H5	0.156	0.6596	0.9329	0.046*	
C6	0.4078 (17)	0.5367 (16)	0.8218 (9)	0.032 (2)	
H6	0.3496	0.476	0.7848	0.038*	
C7	0.251 (2)	0.870 (2)	1.0495 (9)	0.044 (3)	
N3	0.1570 (19)	0.9743 (18)	1.1197 (10)	0.057 (3)	
O1	0.6745 (10)	0.3933 (10)	0.4281 (6)	0.0253 (15)	
O2	0.9632 (11)	0.3531 (12)	0.4658 (6)	0.0303 (17)	
C8	0.8580 (15)	0.2890 (15)	0.4258 (8)	0.0216 (19)	
C9	0.9546 (18)	0.0876 (19)	0.3768 (11)	0.041 (3)	
H9A	1.1009	0.0496	0.3529	0.062*	
H9B	0.8942	0.0826	0.3188	0.062*	
H9C	0.9315	−0.0005	0.4269	0.062*	
O3	0.8051 (11)	0.7257 (11)	0.3578 (6)	0.0280 (16)	
O4	0.4720 (12)	0.8727 (14)	0.3943 (7)	0.042 (2)	
C10	0.6369 (18)	0.8350 (18)	0.3345 (9)	0.032 (2)	
C11	0.642 (2)	0.916 (2)	0.2316 (10)	0.047 (3)	
H11A	0.6577	1.0367	0.2353	0.07*	
H11B	0.5144	0.9398	0.2104	0.07*	
H11C	0.756	0.8238	0.1818	0.07*	

### Atomic displacement parameters (Å^2^)

**Table:**

	*U*^11^	*U*^22^	*U*^33^	*U*^12^	*U*^13^	*U*^23^
Pb1	0.0163 (3)	0.0203 (3)	0.0214 (3)	−0.00385 (19)	−0.00553 (16)	−0.00232 (16)
N1	0.028 (4)	0.015 (4)	0.030 (4)	−0.001 (4)	−0.002 (3)	−0.004 (3)
N2	0.040 (5)	0.073 (9)	0.057 (7)	−0.024 (5)	0.005 (5)	−0.034 (6)
C1	0.030 (4)	0.021 (5)	0.022 (4)	−0.003 (4)	−0.004 (3)	0.001 (3)
C2	0.032 (4)	0.020 (5)	0.027 (4)	−0.002 (4)	−0.012 (3)	0.004 (3)
C3	0.036 (5)	0.033 (6)	0.033 (5)	−0.006 (4)	−0.009 (4)	−0.004 (4)
C4	0.034 (5)	0.029 (6)	0.028 (5)	−0.001 (4)	−0.006 (4)	0.001 (4)
C5	0.033 (5)	0.041 (6)	0.029 (5)	−0.006 (5)	−0.001 (4)	−0.003 (4)
C6	0.033 (4)	0.023 (5)	0.032 (5)	−0.007 (4)	−0.001 (3)	−0.003 (4)
C7	0.044 (7)	0.051 (9)	0.029 (6)	−0.013 (7)	−0.002 (5)	−0.011 (6)
N3	0.062 (8)	0.050 (8)	0.047 (7)	−0.014 (7)	−0.002 (6)	−0.016 (6)
O1	0.017 (3)	0.013 (3)	0.042 (4)	0.000 (3)	−0.013 (3)	0.002 (3)
O2	0.017 (3)	0.034 (5)	0.036 (4)	−0.006 (3)	−0.006 (3)	−0.011 (3)
C8	0.021 (4)	0.020 (4)	0.022 (4)	−0.006 (3)	−0.007 (3)	0.005 (3)
C9	0.027 (5)	0.028 (5)	0.063 (8)	−0.002 (4)	−0.014 (5)	−0.019 (5)
O3	0.023 (3)	0.028 (4)	0.031 (4)	−0.008 (3)	−0.007 (3)	0.002 (3)
O4	0.023 (3)	0.048 (6)	0.043 (4)	−0.001 (4)	−0.012 (3)	0.011 (4)
C10	0.029 (4)	0.036 (6)	0.033 (5)	−0.014 (4)	−0.012 (3)	−0.004 (4)
C11	0.060 (8)	0.042 (8)	0.043 (5)	−0.021 (6)	−0.022 (5)	0.011 (5)

### Geometric parameters (Å, º)

**Table:**

Pb1—O3	2.431 (7)	C4—C7	1.449 (17)
Pb1—O2	2.485 (8)	C5—C6	1.358 (15)
Pb1—O1	2.505 (7)	C5—H5	0.95
Pb1—O2^i^	2.635 (7)	C6—H6	0.95
Pb1—O4	2.667 (8)	C7—N3	1.146 (16)
Pb1—O1^ii^	2.727 (7)	O1—C8	1.255 (12)
Pb1—N1	2.906 (10)	O1—Pb1^ii^	2.727 (7)
N1—C1	1.403 (13)	O2—C8	1.271 (12)
N1—H1A	0.88 (2)	O2—Pb1^i^	2.635 (7)
N1—H1B	0.87 (2)	C8—C9	1.505 (15)
N2—C2	1.398 (15)	C9—H9A	0.98
N2—H2A	0.88 (2)	C9—H9B	0.98
N2—H2B	0.88 (2)	C9—H9C	0.98
C1—C6	1.409 (15)	O3—C10	1.279 (13)
C1—C2	1.430 (15)	O4—C10	1.239 (14)
C2—C3	1.373 (15)	C10—C11	1.500 (18)
C3—C4	1.382 (17)	C11—H11A	0.98
C3—H3	0.95	C11—H11B	0.98
C4—C5	1.393 (17)	C11—H11C	0.98
			
O3—Pb1—O2	77.0 (3)	C4—C3—H3	118.7
O3—Pb1—O1	78.6 (3)	C3—C4—C5	120.0 (11)
O2—Pb1—O1	52.0 (2)	C3—C4—C7	119.9 (12)
O3—Pb1—O2^i^	75.3 (2)	C5—C4—C7	120.0 (11)
O2—Pb1—O2^i^	64.3 (3)	C6—C5—C4	118.5 (11)
O1—Pb1—O2^i^	114.8 (2)	C6—C5—H5	120.7
O3—Pb1—O4	50.8 (2)	C4—C5—H5	120.7
O2—Pb1—O4	117.2 (3)	C5—C6—C1	122.9 (11)
O1—Pb1—O4	82.3 (3)	C5—C6—H6	118.6
O2^i^—Pb1—O4	119.7 (3)	C1—C6—H6	118.6
O3—Pb1—O1^ii^	119.9 (2)	N3—C7—C4	177.7 (16)
O2—Pb1—O1^ii^	108.4 (2)	C8—O1—Pb1	93.8 (6)
O1—Pb1—O1^ii^	63.9 (3)	C8—O1—Pb1^ii^	134.3 (6)
O2^i^—Pb1—O1^ii^	162.4 (3)	Pb1—O1—Pb1^ii^	116.1 (3)
O4—Pb1—O1^ii^	77.9 (3)	C8—O2—Pb1	94.3 (6)
O3—Pb1—N1	147.4 (2)	C8—O2—Pb1^i^	147.4 (7)
O2—Pb1—N1	70.6 (3)	Pb1—O2—Pb1^i^	115.7 (3)
O1—Pb1—N1	83.9 (3)	O1—C8—O2	119.8 (9)
O2^i^—Pb1—N1	87.6 (3)	O1—C8—C9	120.6 (9)
O4—Pb1—N1	152.5 (3)	O2—C8—C9	119.6 (9)
O1^ii^—Pb1—N1	74.7 (2)	C8—C9—H9A	109.5
C1—N1—Pb1	107.9 (6)	C8—C9—H9B	109.5
C1—N1—H1A	108 (9)	H9A—C9—H9B	109.5
Pb1—N1—H1A	109 (9)	C8—C9—H9C	109.5
C1—N1—H1B	106 (9)	H9A—C9—H9C	109.5
Pb1—N1—H1B	119 (9)	H9B—C9—H9C	109.5
H1A—N1—H1B	107 (5)	C10—O3—Pb1	98.9 (7)
C2—N2—H2A	128 (10)	C10—O4—Pb1	88.8 (7)
C2—N2—H2B	123 (10)	O4—C10—O3	121.5 (11)
H2A—N2—H2B	106 (5)	O4—C10—C11	119.8 (11)
N1—C1—C6	120.8 (10)	O3—C10—C11	118.6 (11)
N1—C1—C2	120.7 (10)	C10—C11—H11A	109.5
C6—C1—C2	118.1 (10)	C10—C11—H11B	109.5
C3—C2—N2	122.3 (11)	H11A—C11—H11B	109.5
C3—C2—C1	117.7 (10)	C10—C11—H11C	109.5
N2—C2—C1	120.0 (10)	H11A—C11—H11C	109.5
C2—C3—C4	122.7 (11)	H11B—C11—H11C	109.5
C2—C3—H3	118.7		
			
Pb1—N1—C1—C6	91.4 (11)	C2—C1—C6—C5	−3.7 (18)
Pb1—N1—C1—C2	−81.2 (10)	Pb1—O1—C8—O2	−3.0 (10)
N1—C1—C2—C3	177.1 (10)	Pb1^ii^—O1—C8—O2	−135.8 (8)
C6—C1—C2—C3	4.2 (16)	Pb1—O1—C8—C9	176.5 (10)
N1—C1—C2—N2	−4.3 (17)	Pb1^ii^—O1—C8—C9	43.6 (15)
C6—C1—C2—N2	−177.1 (12)	Pb1—O2—C8—O1	3.0 (10)
N2—C2—C3—C4	177.4 (13)	Pb1^i^—O2—C8—O1	−154.9 (9)
C1—C2—C3—C4	−4.0 (18)	Pb1—O2—C8—C9	−176.4 (10)
C2—C3—C4—C5	3.0 (19)	Pb1^i^—O2—C8—C9	26 (2)
C2—C3—C4—C7	−175.9 (12)	Pb1—O4—C10—O3	2.1 (11)
C3—C4—C5—C6	−2.2 (19)	Pb1—O4—C10—C11	−178.9 (11)
C7—C4—C5—C6	176.7 (12)	Pb1—O3—C10—O4	−2.3 (13)
C4—C5—C6—C1	2.7 (19)	Pb1—O3—C10—C11	178.7 (10)
N1—C1—C6—C5	−176.6 (11)		

Symmetry codes: (i) −*x*+2, −*y*+1, −*z*+1; (ii) −*x*+1, −*y*+1, −*z*+1.

### Hydrogen-bond geometry (Å, º)

**Table:**

*D*—H···*A*	*D*—H	H···*A*	*D*···*A*	*D*—H···*A*
N1—H1*A*···O4^ii^	0.88 (2)	2.46 (4)	3.310 (14)	164 (12)
N1—H1*B*···O3^i^	0.87 (2)	2.40 (8)	3.139 (12)	143 (11)
N2—H2*A*···O3^i^	0.88 (2)	2.25 (9)	3.044 (14)	150 (16)
N2—H2*B*···N3^iii^	0.88 (2)	2.62 (11)	3.355 (18)	142 (14)

Symmetry codes: (i) −*x*+2, −*y*+1, −*z*+1; (ii) −*x*+1, −*y*+1, −*z*+1; (iii) −*x*+1, −*y*+2, −*z*+2.
